# An OCT-A Analysis of the Importance of Intermediate Capillary Plexus in Diabetic Retinopathy: A Brief Review

**DOI:** 10.3390/jcm13092516

**Published:** 2024-04-25

**Authors:** Charbel Haddad, Manon Baleine, Elie Motulsky

**Affiliations:** Department of Ophthalmology, Hôpital Universitaire de Bruxelles, Erasme Hospital, 1070 Brussels, Belgium; charbel.haddad@ulb.be (C.H.); manon.baleine@ulb.be (M.B.)

**Keywords:** intermediate capillary plexus, optical coherence tomography-angiography, diabetic retinopathy

## Abstract

Optical coherence tomography-angiography is a technique that allows us to non-invasively study in vivo the different retinal vascular networks. This allows a deeper understanding of retinal capillary anatomy and function, in addition to the pathophysiologic changes encountered in diverse diseases. The four retinal capillary layers have different anatomies and functions, implying distinct adaptation and roles in the course of the diseases. Diabetic retinopathy is the leading cause of blindness in working-age adults. Several studies have evaluated how each retinal capillary layer is specifically affected according to the stage of the disease. Unfortunately, too few studies have considered the intermediate capillary plexus as a separate layer, as it has often been incorporated in another layer. In this review, we shed light on the potential role the intermediate capillary plexus plays in the physiopathology of diabetic retinal disease as well as its potential use in grading diabetic retinopathy and its clinical added value in estimating the disease prognosis.

## 1. Introduction

**Figure 1:**
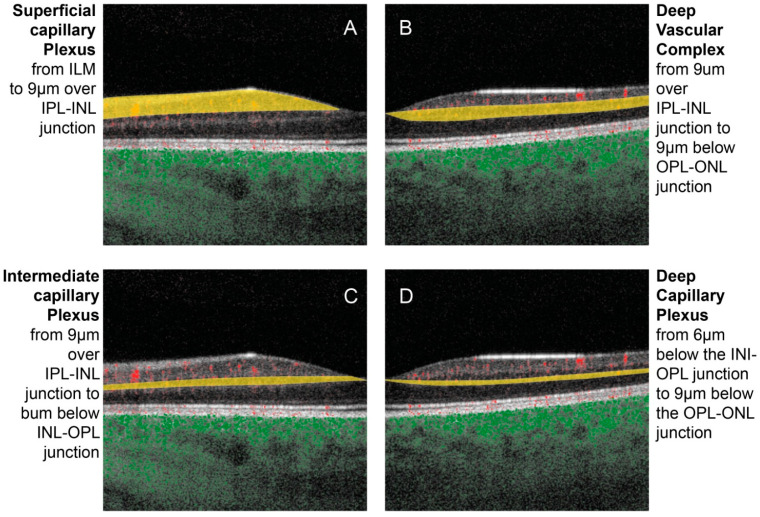
Color-coded B-scan optical coherence tomography-angiography of the macular region using a PLEX^®^ Elite 9000 (Carl Zeiss Meditec Inc., Dublin, CA, USA). The structural retina is depicted in grayscale while the retinal and choroidal vascular flow are in red (the image was collected at Erasme Hospital). (**A**): The superficial capillary plexus in yellow is contained between the inner limiting membrane (ILM) and 9 μm above the inner plexiform layer–inner nuclear layer (IPL-INL) junction. (**B**) shows the deep vascular complex in yellow composed of the combination of the intermediate and deep capillary plexuses. (**C**) shows the intermediate capillary plexus in yellow contained between 9 μm above and 6 μm below the INL-OPL junction. (**D**) shows the deep capillary plexus in yellow going from 6 μm below the INL-OPL junction to 9 μm below the OPL-ONL junction. ILM: internal limiting membrane, IPL: inner plexiform layer, INL: inner nuclear layer, OPL: outer plexiform layer, ONL: outer nuclear layer.

**Figure 2:**
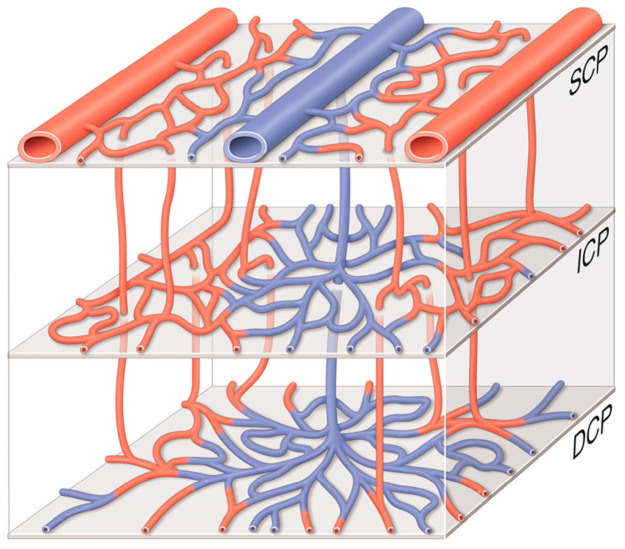
The three retinal capillary layers. The ICP is supplied by arteriolar branches coming from the SCP. The venous outflow of the ICP and DCP forms a single axis that drains into the veins of the SCP. Each layer has its own unique architecture and distribution. DCP: deep capillary plexus, ICP: intermediate capillary plexus, SCP: superficial capillary plexus. Adapted from Garrity et al., courtesy of Pr. Alain Gaudric [9].

The human retina is composed of four layers of capillary plexuses [1]. They differ by their origin, distribution, physiological role, and contribution to the pathophysiology in miscellaneous diseases: a layer located around the optic disc and the temporal arcades in the thick optic nerve fiber layer (ONFL), and three other layers in the ganglion cell layer (GCL) and in the inner and outer plexiform layers (IPL and OPL, respectively) Figure 1 and Figure 2 [1,2].

One of the oldest representations of the human retinal vasculature was done by Wilhelm His in 1880. He described three distinct capillary layers in the macula, with venous drainage going directly from the deepest layer to the superficial one [3]. Decades later, most of our knowledge of the capillary anatomy of the human retina came from fluorescein angiography (FA) images [4,5]. This technique had both the advantages of being dynamic and showing the blood–retinal barrier breakdown in pathological cases. However, the different capillary layers were difficult to distinguish from the background fluorescence. Only the retina’s superficial capillary plexus (SCP) could be inconsistently seen around the fovea and in the periphery [6]. Thus, scientists could not correlate the distinguished layers of capillaries observed in histological studies with clinical photography.

The development of optical coherence tomography-angiography (OCT-A) allowed, for the first time, the detection of the different layers of the retinal capillary plexuses in vivo at a histological resolution. This relatively new non-invasive technology favored numerous works on the human retinal vasculature like never before. Flourishing studies led to multiple interpretations and names for the same structures. While miscellaneous papers started characterizing capillary changes in DR detected by OCT-A, no consensus was reached and the proper separation between each capillary layer was not clear and differed in the work of various teams. Furthermore, these early papers did not differentiate the ICP from the DCP; it was not until 2017 that Campbell et al. suggested a nomenclature, including the SCP, that would be adopted almost everywhere [7,8]. Understanding how a disease affects a specific retinal microvasculature layer is of utmost importance as physiopathological comprehension of a condition might lead to new therapeutics. Frequent retinal diseases such as diabetic retinopathy (DR) or retinal venous occlusion deserve to be investigated at a capillary level using in vivo image technologies.

**Figure 3:**
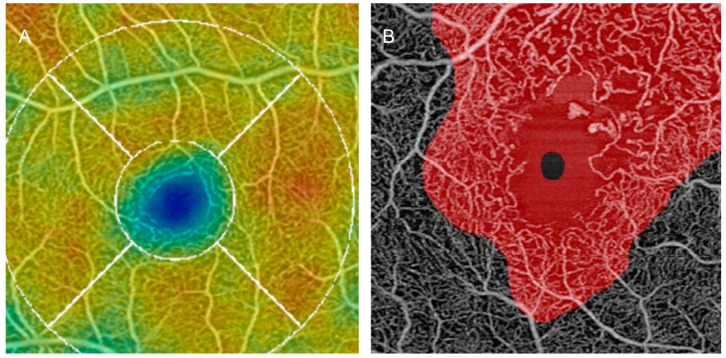
*En-face OCT-A images*, taken with the PLEXElite 9000 OCT-A and analyzed using the ARINetwork Hub (the initial image was collected at Erasme Hospital). Image (**A**) shows a color-graded image centered on the fovea with an ETDRS grid (white curves) based on vessel density in a healthy eye. Image (**B**) is an example of an automated recognition system that marks in red suspicious altered retinal areas on *en-face angiograms*.

The advancement of OCT-A machinery and software has allowed clearer images with higher resolutions and a wider field of view. Also, analyses algorithms that are being developed, like the ARINetwork hub by Zeiss, can give us various in-depth photographic analyses of *en-face images*. Most of the current algorithms focus on extracting the data contained in the images acquired by the OCT-A and in revealing pertinent data presentations such as vessel density or flow voids depending on the studied disease. Current color grading in the native PLEXElite software instrument (software version 2.1.0.55513) is based on the layer depth. An unusual color change will draw the attention of the clinician to quickly detect suspicious areas of disease Figure 3.

DR is the single most common microvascular complication of type 1 and type 2 diabetes [10]. It can lead to progressive vision loss and even blindness [11]. Chronic hyperglycemia alters the capillaries’ permeability and leads to progressive vascular occlusion ending in retinal ischemia. Despite advancements in retinal imaging and therapeutic options, DR remains one of the top causes of blindness in working-age adults. The global DR burden is expected to remain high through 2045 [12,13]. Since the development of OCT-A, a large number of teams have studied the changes occurring in each retinal capillary layer during DR. Vessel density (VD), one of the most commonly studied parameters, was found to decrease with increased DR severity. Interestingly, the rate of VD decrease was different when studying each layer separately [14]. Another prospective study found that low VD of the deep capillary plexus (DCP) was associated with an increased risk of DR progression [15]. As in other studies, the retina was separated into two retinal capillary plexuses, a superficial and a deep one. Too often, the intermediate plexus is incorporated into the superficial one by the software algorithms. Another significant parameter is the geometric perfusion deficit (GPD), a new OCT-A-based measurement defined as an area with a distance of more than 30 μm from the nearest vessel [16]. Nesper et al. investigated the clinical relevance of the GPD in the DR using OCT-A images. They aimed to detect clinically referable eyes with DR (eyes with moderate non-proliferative DR or worse or eyes with diabetic macular edema). The fovea was included in their GPD measurement (GPDf) and considered as a separate parameter. They found that the GPDf of the DCP was highly sensitive for identifying referable eyes [17]. Many OCT-A parameters such as GPD and GPDf are emerging and might become a standard non-invasive biomarker for DR.

All these data emphasize how pathological changes of the retinal capillary layers might be specifically affected by the disease. Onishi et al. divided the retinal vascularization into three different layers and compared the adjusted flow index (AFI) of the SCP, intermediate capillary plexus (ICP), and DCP. They found that in contrast to the AFI of the ICP and of the DCP, the AFI of the SCP did not significantly differ between eyes without DR and eyes with various degrees of DR [18]. Compared to the other layers, they also showed that the percentage of non-perfused areas was lower for the ICP in diabetic patients, which might also point out the importance of this resistant intermediate layer in preserving the nurturing of the macular area while other vascular layers begin to fail.

The ultimate goal of these technological advancements is earlier diagnosis, grading, prognosis, and management/treatment/prevention of DR: early alterations that sometimes precede overt DR can be crucial in early detection of patients at risk of developing DR. In the past, it was believed that vascular abnormalities are the earliest signs of this disease, but recent studies have shown that neuronal abnormalities can be present long before DR can be clinically detected, and clinical correlations between these alterations and OCT-A parameters have been described [19]. 

## 2. The Intermediate Capillary Plexus

Outside of the papillary region, the retinal capillary vasculature is divided into three separate layers [Table 1] [7]. The ICP is defined as the capillary plexus that starts at the inner two-thirds of the IPL and reaches the limit between the inner nuclear layer (INL) and the OPL. It develops in the perinatal period by sprouting from the DCP, driven by the angiogenic stimuli of the amacrine cells [20]. It is supplied by vertical anastomoses coming from the SCP [Table 1] [7]. In contrast to the SCP where the capillaries are distributed in the volume between the NFL and GCL, the ICP forms a narrow laminar plane [21].

At around 6 to 7 mm temporally to the fovea, the ICP and DCP start to merge to form a single capillary plexus due to the thinning of the GCL which does not require two separate capillary supplies. The density of these merged plexuses is higher than the one of the SCP found in the peripheral retina. Interestingly, the SCP density is higher than the density of the ICP and DCP in the peripapillary, parafoveal, and perifoveal locations [7]. Some authors have hypothesized that the ICP vanishes instead of merging with the DCP, seeing as the need for a third plexus becomes less and less vital as we move away from the macula [2,7].

In a trial studying the effect of ocular hypertension on retinal ganglion cells and capillary plexuses, Pitale et al. found that over a period of 2 weeks, the intermediate plexus lost more capillary junctions and capillary density than the two other capillary layers [22]. Diabetic and hypertensive studies suggest that the intermediate complex could also be an adaptative blood supply for the superficial and deep complexes as it first adjusts prior to other capillary networks in the early stage of the disease. Also, the disappearance of the ICP layer going towards the periphery could potentially explain why the peripheral retina is usually first affected in diabetes: the diminishing need for oxygen supply in the thinning retina remains less important than the decrease in the oxygen supply. As mentioned in Lavia et al.’s work, when the ICP vanishes, the interplexus distance tends to increase despite the thinning retina [2]. As Silva et al.’s work showed, patients with predominantly peripheral diabetic lesions (PPL)—a region where the ICP is absent—were at higher risk of DR progression compared to people without PPL [23]. All the aforementioned arguments converge on the potentially relevant role of the ICP in the development of DR. As the vascular parameters develop, the disease’s progression risk will become more and more accurate, suggesting that the ICP might one day provide a visual prognosis to our patients.

## 3. OCT-A Image of the ICP

Compared to fluorescein angiography, OCT-A is a relatively new technique of retinal vascular imaging that uses an infrared laser beam that is reflected by the posterior part of the eye. It images different consecutive horizontal B-scans at each spatial location within the area visualized. An algorithm compares all the acquired images. Based on the red blood cells’ motion, the system reconstructs the retinal vessels and measures a relative blood flow speed [24]. New algorithms can generate high-resolution images and allow for numerous measurements including the VD, GPD, and AFI [2,24,25,26]. AFI is a parameter that correlates linearly with flow velocities found in the retina. However, most of the OCT-A software today solely distinguishes the SCP and DCP. Additional retinal depth segmentation is required for the OCT-A to discriminate the three different layers [26].

In the beginning, OCT-A technology had a lot of drawbacks due to the presence of unprecedented artifacts such as vessel projection, retinal vessels duplication, etc. [27]. Projection artifacts and decorrelation trails are caused by moving red blood cells in the SCP that project their shadows to the deeper layers, which are interpreted as motion by OCT-A machines, hence blurring the different vascular layers and merging them [27]. Higher resolution images that could decrease artifacts would, thus, require higher acquisition times [28]. In a paper published in 2021, Hormel et al. summarized the different techniques and algorithms developed to limit, but not completely remove, the different types of artifacts encountered using OCT-A [21]. Various softwares recommend a predefined segmentation, but we still have to manually customize the segmentations to measure the ICP parameters. Though this procedure reduces the risk of misidentification of each retinal layer, it is time-consuming and not sustainable in our daily practice. Also, compared to the SCP, both the ICP and DCP, being deeper layers, tend to have higher image artifacts due to the lower flow signal, which often leads to truncated vessels on *en-face images*. Researchers are using deep-learning networks like the Deep Capillary Angiogram Reconstruction Network developed by Goa et al. to obtain images with reduced noise intensity and improved continuity of vessels on angiograms [28]. Each technological advancement enhances a different type of artifact or noise, and thus, should promptly be implemented in clinical OCT-A software. Clinically, DR is often characterized by peripheral ischemia. This means that the usual 3 × 3 mm and 6 × 6 mm OCT-A images were perhaps missing some DR lesions and could affect patient management. The relatively small area scanned by the OCT-A was due to a high image acquisition time, limiting our peripheral vascularization status, often insulted during ischemic DR. This issue has been solved with the development of the swept-source OCT-A imaging techniques that permit faster and deeper acquisition, higher resolution, and wider images reaching sizes of 12 × 12 mm for the PLEXElite 9000 (Carl Zeiss Meditec, Inc., Dublin, CA, USA) or 20 × 23 mm for the Xephilio OCT-S1 (Canon, Tokyo, Japan). Modern software developed by many manufacturers now offer montage options where smaller acquisitions are needed and can be put together to reconstruct a single wider retinal image. This being said, the ICP is a layer that is mainly present around the fovea: as described by Lavia et al., the ICP tends to merge with the DCP at around 6 mm from the fovea to form a single capillary layer, which implies that wider imaging techniques are probably useless in providing consistent information regarding the ICP. 

In addition to OCT-A, newer OCT technologies like photothermal OCT and photoacoustic imaging are being developed [29,30]. These new imaging techniques allow for further molecular visualization and can each offer certain advantages compared to other techniques. Although these developments are still in their early development phases and, thus, not used in clinical practice, the future of patient care should target a multimodal analysis of disease processes, as each technique offers specific insights. 

## 4. ICP and Diabetic Retinopathy

In OCT-A studies, it has been shown that the ICP is unevenly affected in DR compared to the other layers and it is also differently altered according to DR severity. The ICP is at increased risk compared to other layers for the formation of micro-aneurysms and capillary loops [18,31,32].

Eyes with mild non-proliferative DR (NPDR) had a significantly lower VD in the ICP compared to eyes with no DR. This difference was also noticeable in eyes with very early stages of DR [14]. Remarkably, these VD changes in ICP were less significant in advanced stages of DR. Interestingly, in the early stages of DR, the VD of the ICP significantly changes, whilst at later DR stages, as the disease progresses, the VD of the ICP exhibits less changes uncorrelated to the DR stage. This might underline the importance of the ICP in the early stages of the disease. The ICP, being the first to be affected by DR changes, might play a role in regulatory vascularization of other capillary layers. On the contrary, Zhang et al. compared the eyes of people with early NPDR with controls and found that the ICP had fewer avascular areas compared to the SCP and the DCP [33]. Withal, these results have been generated using a 3 × 3 mm area picture focused on the macula, whereas diabetic retinal ischemia might spread in an inhomogeneous pattern affecting any retinal region including the periphery. 

In their paper on advanced retinal vascular imaging in diabetes, Ong et al. concluded that as the DR severity progresses, the blood flow in the ICP and DCP worsens, while the flow in the SCP remains the same or increases [34]. These ambiguous responses to the same disease could be explained by an auto-regulatory dysfunction due to impaired neurovascular coupling that occurs in diabetes [35]. When studying GPD, Nesper et al. included the ICP with the DCP in their paper. This segmentation showed that this “inclusive DCP” allowed for the detection of referable eyes. This additional grading method helps doctors in screening for DR [17]. Further custom segmentation that distinguishes the ICP from the DCP could provide further insight into the role of each layer during DR at each stage of the disease. 

Many OCT-A parameters have thus been retrospectively associated with DR severity. Albeit, to the best of our knowledge, only a few prospective studies have evaluated how OCT-A parameters could predict the risk of progression of DR. One of these prospective studies, conducted by Sun et al., regrettably, did not divide the retinal vasculature into three separate layers and included the ICP into the SCP [15]. In their work, they found that the decreased VD of the SCP is associated with an increased risk of macular edema development, while the decreased VD of the DCP was associated with an increased risk of progression of DR (9]. Another prospective study conducted by You et al. in 2020 aimed to find an association between OCT-A quantifiable parameters and DR severity, progression, and treatment requirement after a one-year follow-up. They used a 3 × 3 mm scan centered on the macula and divided the retinal capillary networks into three separate layers. At baseline, they found that the extrafoveal avascular areas (EAA) of all the layers were correlated with DR severity. The EAA of the DCP was also correlated with worse visual acuity. A multivariate logistic regression model found that disease progression was associated with the EAA of the SCP. Another multivariate model found an association between treatment requirements during the one-year follow-up and the EAA of the DCP. When separating treatment-naïve eyes from the rest, the EAA of the ICP was significantly correlated with treatment requirement [36]. This difference between treatment-naïve and treated eyes could be explained by the fact that treated eyes have usually reached more severe stages of DR, and as already mentioned previously, the ICP is affected the most at the beginning of the retinal insult occurring in diabetes [14]. You et al.’s work focused on a 3 × 3 mm central scan, whilst diabetic ischemic lesions usually start at the periphery.

Midena et al. showed that early alterations in the electro-retinogram oscillatory potential (OP), which is sensitive to changes in retinal circulation, were detected in patients with diabetes with no clinical or in very early stages of DR [19,37]. Their OCT-A study showed that OP alterations were associated with shorter vessels in the SCP and dilated vessels in the ICP: this supports the argument that the ICP plays a role in regulating the superficial vasculature of the retina in the beginning of DR. Also, it highlights the potential use of OCT-A images as a biomarker detecting early signs of neurovascular diabetic dysfunction [19]. Borrelli et al. found similar results in their work showing that the ICP in patients with non-proliferative DR had a higher vessel diameter index compared to healthy controls [38].

## 5. Conclusions

DR is a serious complication of diabetes and is one of the most common causes of vision loss. The first clinical symptoms of DR are usually the micro-aneurysms forming in the capillaries of the retina that lead to progressive occlusion of the retinal microcirculation, ending in ischemia and, later on, in dramatic vision loss, often not reversible. The retinal vasculature is divided into three separate layers, outside of the papilla, the SCP, ICP, and DCP. The ICP, which extends from 9 μm above and 6 μm below the INL-OPL junction, is supplied by arterioles originating from the SCP. It forms a narrow plane that extends from the fovea and decreases gradually to fade away at around 8–9 mm away in the peripheral retina. This area is where the ischemic retina can be found in DR. In earlier stages of DR, many teams have found increased vessel diameter index, which underlines a possible regulatory role of the capillaries in early stages of the disease. At the same time, the VD of the ICP tends to decrease in the early DR stage but does not correlate with the disease progression. This arises peculiarly at the early stages of DR, which sheds light on the possible involvement of this intermediate retinal vasculature network in the pathophysiology of the DR. The ICP might play a crucial role during the initiation of DR as it could regulate and adapt its network to minimize other retinal layer damage, similarly to cerebral capillaries where different layers and orders of vessels react differently when faced with ischemic stress. In addition to controlling and stabilizing glycemia in diabetic patients, prompt management and treatment of DR is decisive in preventing its progression to severe stages and vision loss. For that purpose, at-risk patients should be screened in the early stage of DR. Future prospective studies should be conducted to confirm that ICP parameters measured by the OCT-A can effectively predict the risk of progression of DR. So far, multiple cross-sectional studies have underscored the importance of separating human retinal vasculature into three separate layers, each playing a unique role and being irregularly affected in different stages of DR. There would be a compelling interest in further investigating the three retinal capillary layers separately, both in the central and peripheral retina, to discern the importance of their respective parameters in predicting DR progression. This review underlines the potential of ICP analyses utilizing OCT-A in enhancing disease prevention, diagnoses, and outcomes. However, several barriers persist in its implementation into daily clinical practice: prolonged acquisition time, cost, and limitation in detecting vessel leakage. Consequently, it should be regarded as a supplementary tool alongside existing diagnostic modalities for managing DR, rather than a replacement.

## Figures and Tables

**Table 1:** Nomenclature and limits of the different capillary layers found in the neuroretina. NFL: nerve fiber layer, GCL: ganglion cell layer, IPL: inner plexiform layer, INL: inner nuclear layer, OPL: outer plexiform layer, ONL: outer nuclear layer, PR: photoreceptors layer, RPCP: radial peri-papillary capillary plexus, SCP: superficial capillary plexus, ICP: intermediate capillary plexus, DCP: deep capillary plexus, SVC: superficial vascular complex, DVC: deep vascular complex. Adapted from Campbell et al. [7].

Neuroretinal Anatomic Layers	Vascular Nomenclature Correspondence	
NFL	RPCP	SVC
GCL	SCP	
IPL		
	ICP	DVC
INL		
OPL		
	DCP	
ONL		
	Avascular Layers	
PR		

## Data Availability

No new data was created.
